# Bilateral exudative retinal detachments and associated choroidal detachments in a patient on dapsone: a case report

**DOI:** 10.1186/s40942-022-00383-3

**Published:** 2022-05-20

**Authors:** Sarah P. Dugan, Hakan Demirci

**Affiliations:** Kellogg Eye Center, Department of Ophthalmology and Visual Sciences, University of Michigan, 1000 Wall St, Ann Arbor, MI 48105 USA

**Keywords:** Dapsone, Choroidal effusion, Exudative retinal detachment

## Abstract

**Background:**

Dapsone is a synthetic sulfonamide used to treat numerous dermatologic conditions. Ocular side effects have been rarely reported and include retinal necrosis, optic atrophy, and macular infarction. We report the first known case of bilateral choroidal effusions and exudative retinal detachments associated with dapsone use.

**Case presentation:**

A 57-year-old male with a past medical history of testicular seminoma presented with bilateral blurry vision for 2 months. His exam revealed bilateral choroidal effusions with bilateral exudative retinal detachments without evidence of intraocular tumor. The patient had recently been prescribed dapsone for urticarial vasculitis. The patient was instructed to discontinue dapsone and follow-up closely. Interval follow-up of 8 months demonstrated almost complete resolution of the choroidal effusions and retinal detachments with residual pigment epithelium changes after cessation of dapsone. The patient recovered his pre-detachment visual function.

**Conclusions:**

Patients on dapsone who present with new visual complaints should undergo a thorough ophthalmic evaluation given the multiple mechanisms by which dapsone can affect the eye.

## Background

Dapsone is a synthetic sulfonamide with anti-microbial and anti-inflammatory effects used in the treatment of numerous dermatologic conditions, including leprosy, urticarial vasculitis, and dermatitis herpetiformis, among others [1]. As an anti-microbial agent, dapsone inhibits the synthesis of dihydrofolic acid in a manner similar to sulfonamides. Its anti-inflammatory mechanism of action is less understood, but it likely impacts neutrophil function via multiple mechanisms, including through interference with myeloperoxidase, inhibition of neutrophil lysosomal enzymes, and stabilization of lysosomes [2]. Dapsone undergoes enterohepatic circulation after absorption and is metabolized primarily by the liver as well as activated polymorphonuclear leukocytes and mononuclear cells. In the liver, dapsone undergoes both acetylation and hydroxylation to form pharmacologically active metabolites that are thought to be responsible for its therapeutic and adverse effects. Dapsone is distributed to all organs and may remain in certain tissues for up to 3 weeks after cessation of therapy [1]. Ocular side effects of dapsone are uncommon and are often associated with dapsone overdose, though toxicity at therapeutic levels has also been reported. Ocular manifestations of toxicity include retinal necrosis, optic atrophy, and macular infarction [3–6]. To our knowledge, extensive literature search shows no previous reports of bilateral choroidal effusions or retinal detachments associated with dapsone use. Adverse drug event databases, such as the Federal Drug Administration Adverse Events Reporting System and EudraVigilance, also do not contain any reports. Early recognition of detachment and cessation of the offending agent can lead to significant visual recovery. Thus, providers who prescribe dapsone should be aware of this potential side effect. We report what we believe to be the first case of bilateral choroidal effusions and exudative retinal detachments associated with dapsone use.

## Case presentation

**Fig. 1:**
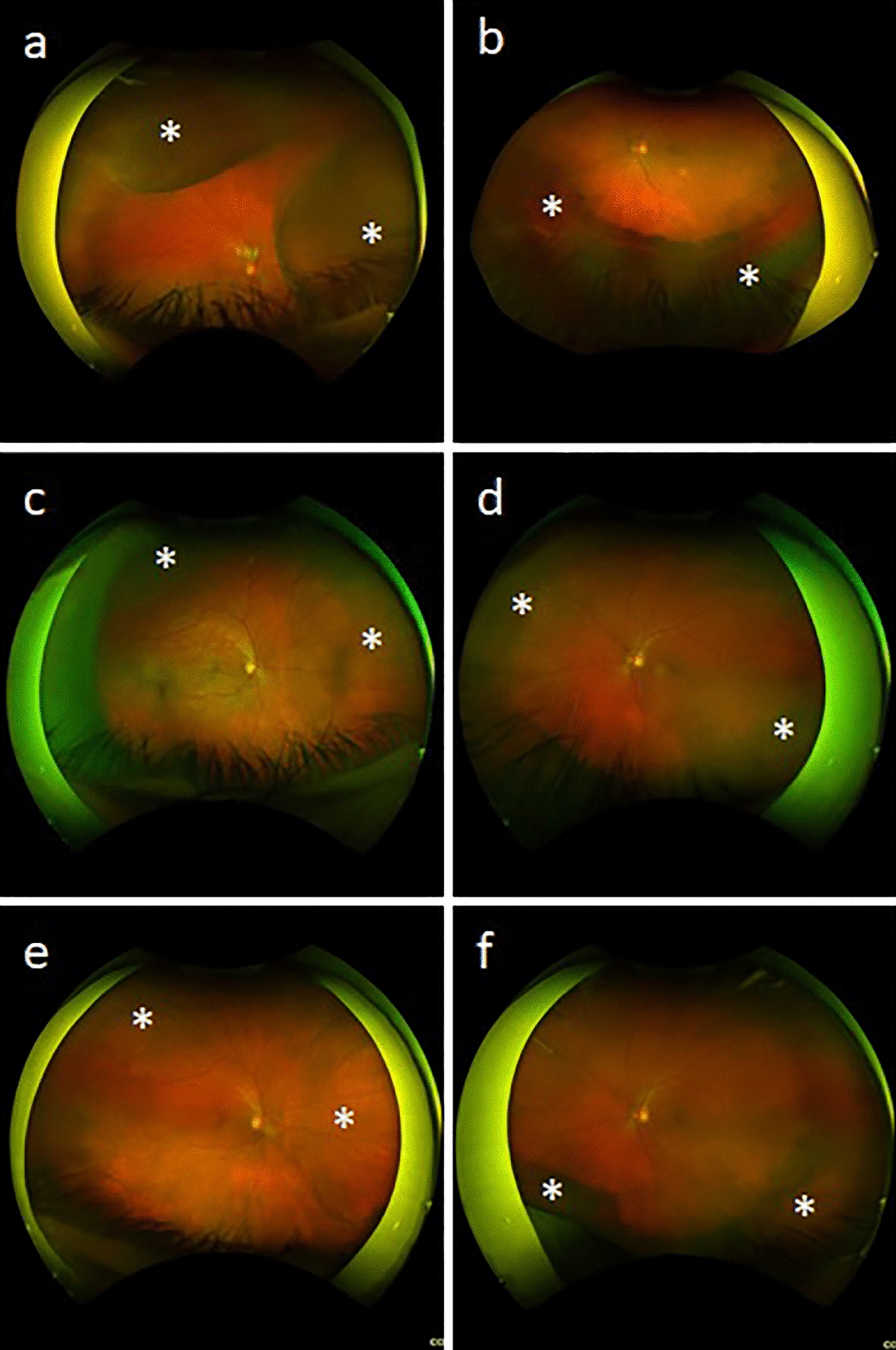
Fundus photography demonstrates choroidal detachment in the patient’s right (**a**) and left (**b**) eye on initial presentation. 3.5 months later the right (**c**) and left (**d**) eyes show improvement after dapsone cessation. The right (**e**) and left (**f**) eyes show near resolution of the choroidal effusions 8 months after initial presentation

**Fig. 2:**
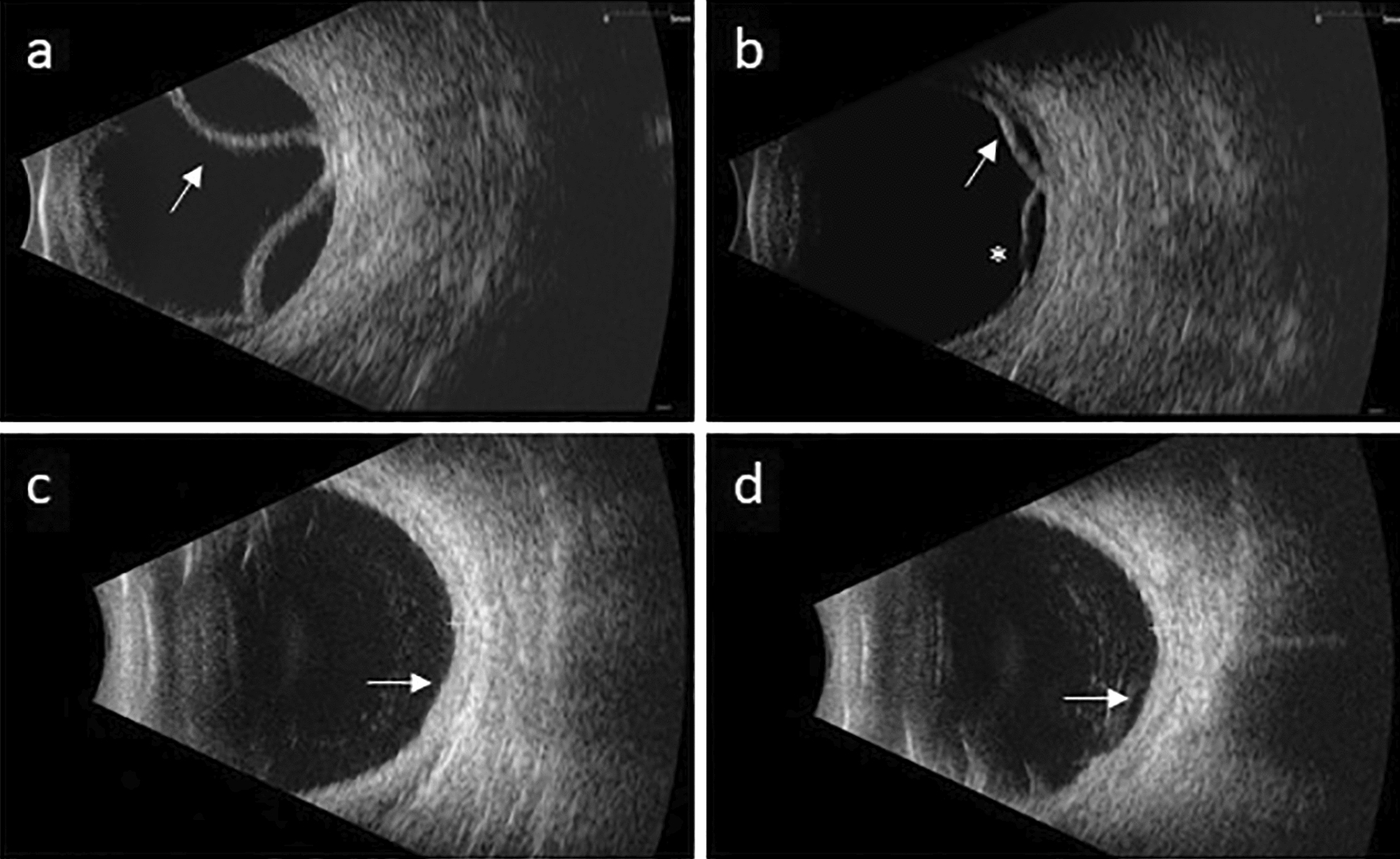
Ultrasound from patient’s initial presentation shows 360° of choroidal effusion in the right (**a**) and left (**b**) eye (arrows). The left (**b**) eye also demonstrates a focal retinal detachment inferiorly (asterisk). Repeat ultrasound 8 months later shows very shallow choroidal detachments in the right (**c**) and left (**d**) eyes (arrows)

**Fig. 3:**
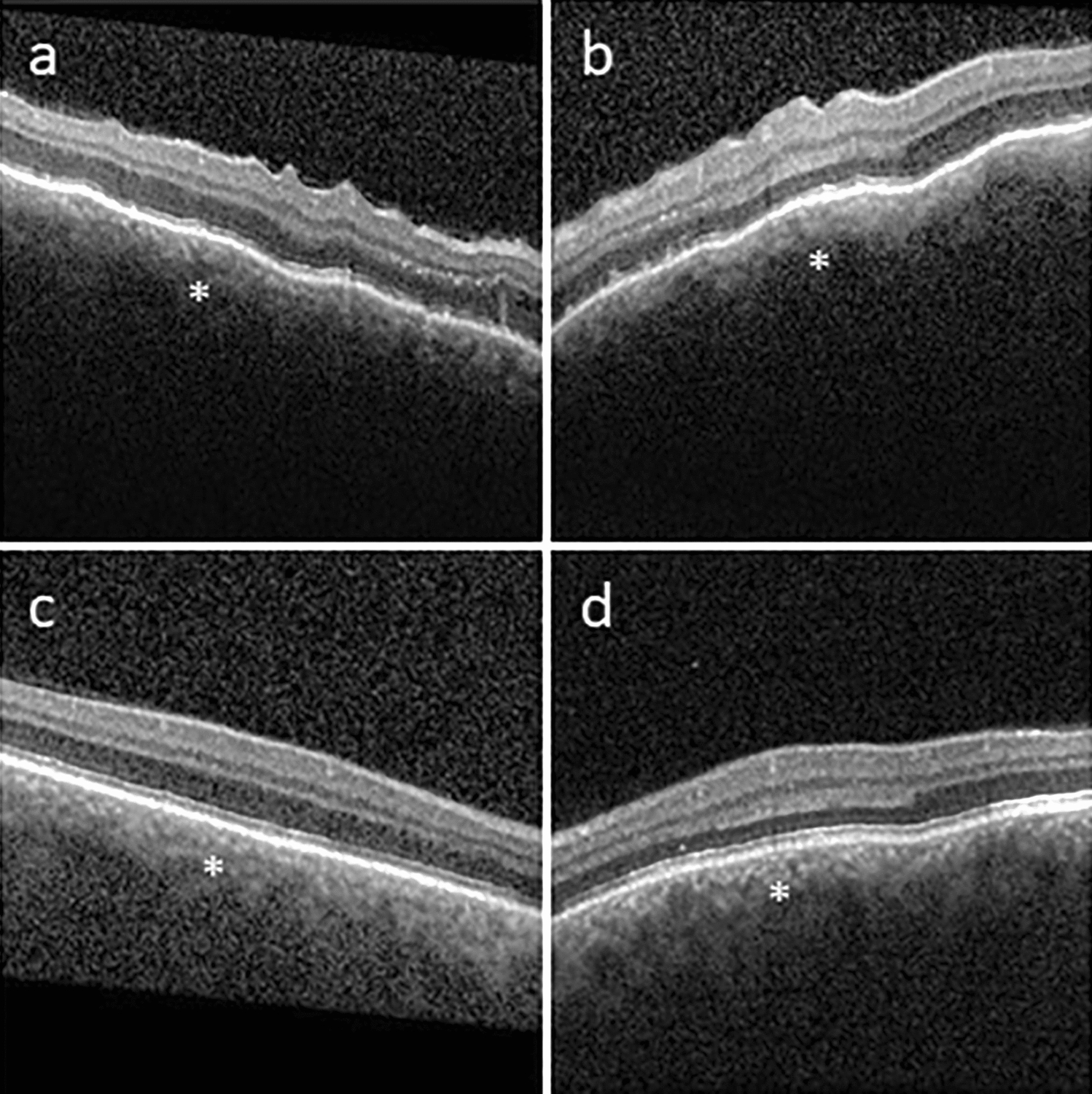
OCT at the time of patient’s initial presentation demonstrates diffuse choroidal and outer nuclear layer thickening/fluid in the right (**a**) and left (**b**) eyes with near complete resolution in the right (**c**) and left (**d**) eyes 8 months later

**Fig. 4:**
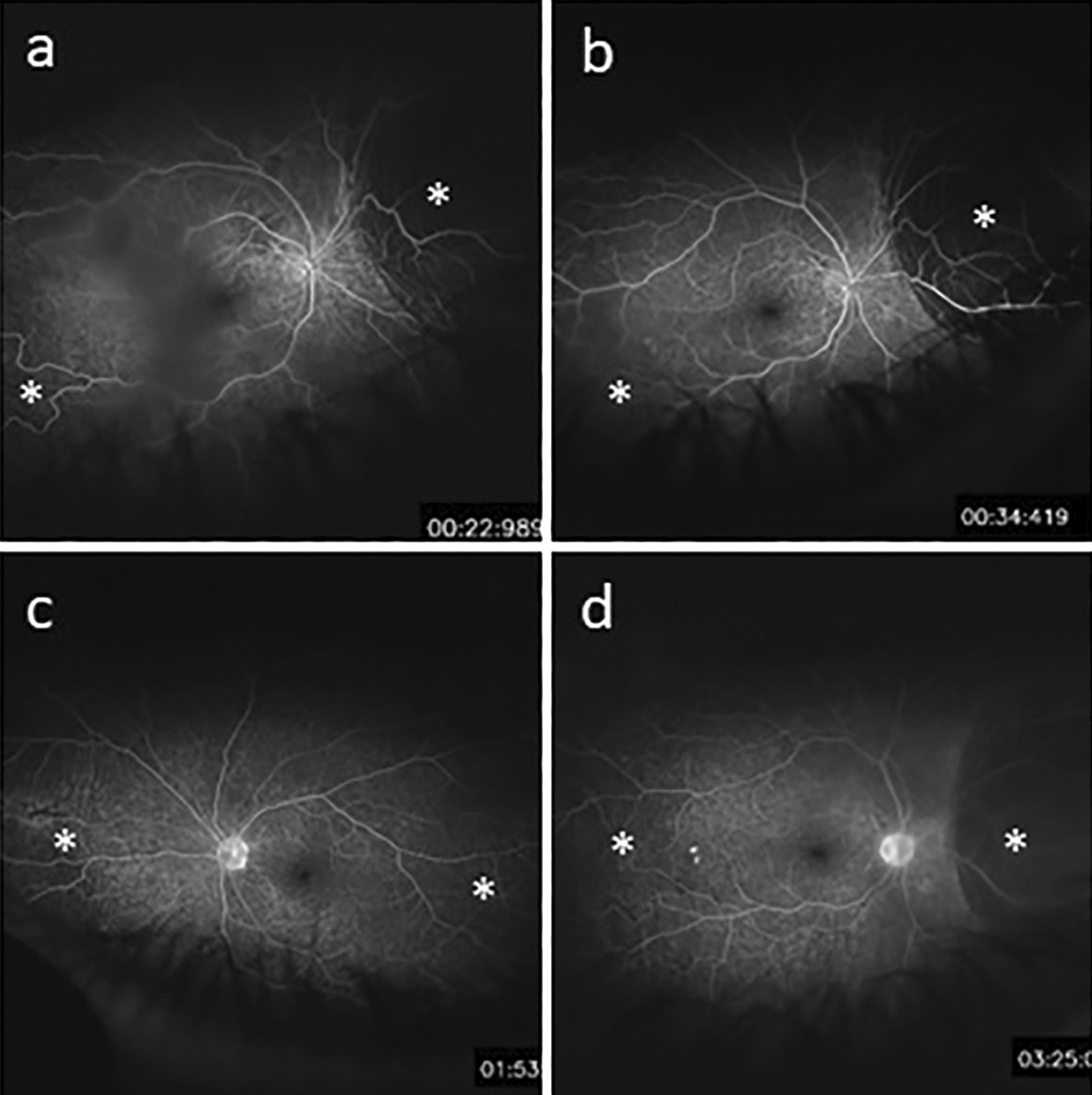
Fluorescein angiography at the time of patient’s initial presentation shows hypofluorescence in the periphery consistent with bilateral choroidal detachments in the arteriovenous phase of the right eye (**a**), venous phase of the right eye (**b**), and the late recirculation phases of the right (**c**) and left (**d**) eyes

A 57-year-old male with a past medical history of controlled rheumatoid arthritis on adalimumab, urticarial vasculitis on prednisone and cetirizine, and testicular seminoma was referred to ocular oncology due to concern for bilateral choroidal metastases. He was diagnosed with testicular seminoma 3 years prior and treated with surgical resection followed by systemic chemotherapy due to retroperitoneal lymph node involvement. He had been in remission for 2 years. He presented with a 2 month history of bilateral blurry vision and photopsias. He had no ophthalmic history, and his only ocular medication was artificial tears. His best-corrected visual acuity (BCVA) was 20/40 in both eyes. Intraocular pressure was 14 mmHg in the right eye and 15 mmHg in the left eye. Slit lamp exam demonstrated diffuse conjunctival hyperemia bilaterally. There were no signs of inflammation in the anterior chamber or vitreous. Fundoscopic exam showed exudative retinal detachments associated with choroidal detachments in both eyes (Fig. 1a, b). There were no retinal or choroidal lesions or signs of vasculitis. B-scan ultrasonography revealed bilateral, 360-degree choroidal effusions with a maximal elevation of 6.7 mm in the right eye and 1.7 mm in the left eye (Fig. 2a, b). Ultrasound of the left eye also showed an exudative retinal detachment (Fig. 2b). The scleral thickness was 0.8 mm in the right eye and 0.9 mm in the left eye. The axial length was 22.7 mm in the right eye and 22 mm in the left eye. Optical coherence tomography (OCT) confirmed diffuse choroidal thickening and exudative retinal detachments bilaterally (Fig. 3a, b). Fluorescein angiography (FA) showed hypofluoresence more prominent in the nasal than temporal periphery of both retinas (Fig. 4a–d). There was no evidence of choroidal metastasis on exam or imaging. Further history revealed that 2 months prior to presentation, the patient had begun taking 50 mg of dapsone daily for his urticarial vasculitis. The patient had previously tested negative for glucose-6-phosphatase dehydrogenase (G6PD) deficiency. Despite the negative G6PD deficiency test, it was thought that dapsone could be contributing to his symptoms given their temporal association with the drug. The patient stopped taking dapsone.

Two weeks after he stopped dapsone, his BCVA improved to 20/20 in both eyes and his exudative retinal detachments and choroidal detachments started to improve bilaterally (Fig. 1c, d). Eight months after stopping dapsone, his BCVA was 20/20 in both eyes. His exudative retinal detachments were completely resolved, and his choroidal detachments were almost completely resolved. There was mild choroidal thickening nasally and temporally as well as residual retinal pigment epithelium changes at the periphery in both eyes (Figs. 1e, f, 2 and 3c and d).

## Discussion

The most commonly reported side effects of dapsone include hemolytic anemia, methemoglobinemia, and gastrointestinal side effects. These side effects are known to be dose-dependent, and to affect patients with G6PD deficiency more severely [1, 7]. However, patients without G6PD deficiency, such as the patient in this case, are also susceptible to the oxidative damage caused by the drug. Methemoglobinemia is also often reported in patients on therapeutic doses of dapsone. The amount of methemoglobin in patients on dapsone does not usually exceed 5−15% of total hemoglobin, however, it is not uncommon for patients to have much higher levels [2, 7]. The hematologic side effects of dapsone have been well-described in the literature and are attributed to dapsone hydroxylamine (DDS-NOH), a pharmacologically active metabolite of dapsone that causes intracellular oxidation [1]. This metabolite is the result of hydroxylation by hepatic cytochrome P-450 enzymes. DDS-NOH rapidly enters the circulation and disrupts the integrity of red blood cells (RBCs) as a result of glutathione depletion and tyrosine phosphorylation of band 3 protein [8]. Sub-hemolytic levels of DDS-NOH may also induce a prothrombotic state in patients on dapsone via the formation of reactive oxygen species [8]. All patients invariably experience some level of hemolysis and methemoglobinemia, although most do not experience clinically significant effects [1].

Previous ocular adverse reactions to dapsone have result in retinal necrosis, optic nerve atrophy, and maculopathy [3–5, 9]. It has been hypothesized that the ocular side effects of dapsone are not due to direct toxicity of the drug, but rather due to ischemia from two distinct mechanisms whose combined effects result in visual changes. Hemolysis causes RBC fragmentation that blocks small vessels in the retina and choroid. Fragmented RBCs are frequently seen in patients taking dapsone, even at therapeutic levels [6]. Accumulation of these cells in small vessels is hypothesized to create a “sludging” effect that causes infarction, although smaller studies have questioned if this occurs at lower doses of dapsone [10]. This obstruction is then exacerbated by dapsone-induced methemoglobinemia. The methemoglobinemia lowers the oxygen-carrying capacity of the blood and diminishes the functionality of the remaining hemoglobin, leading to tissue hypoxia and injury [3–6, 9]. Exudative retinal detachments can be caused by any disruption of the outer blood-retinal barrier. Hypoxic damage to the blood-retinal barrier is uncommon, but can occur when choroidal circulation is dramatically reduced [11]. This may result in ischemic injury with vascular compromise, leading to dysfunction of the retinal pigment epithelial pump mechanism and subsequent exudative detachment [12]. It is thus possible that vascular obstruction combined with some component of methemoglobinemia could have contributed to this patient’s ocular findings.

It is well-described in the literature that sulfa drugs may cause new-onset choroidal effusions, and this often presents as acute angle closure glaucoma (AACG) [13]. The mechanism of how sulfonamides cause choroidal effusions has not been elucidated, but idiosyncratic hypersensitivity reactions have been proposed in previous cases [13, 14]. Although dapsone has not previously been associated with spontaneous choroidal effusions with resulting AACG, we felt it important to consider given its status as a sulfa drug. Other potential etiologies of choroidal effusion include hypotony and pro-inflammatory conditions, however, this patient’s intraocular pressures were normal, and he had no signs of inflammation on his exam despite his comorbid autoimmune conditions. Urticarial vasculitis often has ophthalmic manifestations, but these most commonly present as episcleritis, uveitis, or conjunctivitis, not choroidal or exudative retinal detachments [15]. Adalimumab and cetirizine are not known to be associated with choroidal or retinal detachments. An association between systemic corticosteroid therapy and bilateral exudative retinal detachments has been observed, however, this is less likely to be the cause of this patient’s presentation [16]. The patient in our case had been on prednisone for many years and continues to take the medication. The timing of symptom development and resolution does not coincide with any alterations in prednisone therapy but does align with initiating and stopping dapsone.

This case demonstrates the importance of ophthalmic evaluation in patients on dapsone who present with new visual complaints. If a dapsone adverse event is suspected, clinicians should consider further laboratory workup to evaluate for hemolysis, including a complete blood count with reticulocyte count, a methemoglobin level, and a G6PD test if not previously performed. Discontinuing the drug early, as in this case, may result in near complete return of visual function. This might be important especially for patients on dapsone who are at higher risk from hemolytic side effects, such as patients with G6PD deficiency or sickle cell anemia, or those who are at higher risk for exudative retinal detachment or choroidal detachment, such as patients with systemic inflammatory disorders.

## Conclusions

In summary, we present a novel case of a previously unreported ocular side effect associated with dapsone. Patients on dapsone who present with new visual complaints should undergo a thorough ophthalmic evaluation given the multimodal mechanisms of ischemia and the potential for significant visual recovery if the offending drug is stopped early.

## Data Availability

Not applicable.

## Abbreviations

AACG: Acute angle closure glaucoma
DDS-NOH: Dapsone hydroxylamine
BCVA: Best-corrected visual acuity
G6PD: Glucose-6-phosphatase dehydrogenase
OCT: Optical coherence tomography
RBC: Red blood cell
